# Nobiletin alleviates endometriosis via down-regulating NF-κB activity in endometriosis mouse model

**DOI:** 10.1042/BSR20180470

**Published:** 2018-06-21

**Authors:** Xin Wei, Xu Shao

**Affiliations:** Liaocheng People’s Hospital, Dongchang West Road #67, Liaocheng 252000, China

**Keywords:** endometriosis, endometriosis mouse model, IκB kinase, nobiletin, nuclear factor-kappa B

## Abstract

Nobiletin exhibits protective potential on inflammation and inhibits the activation of transcription factors nuclear factor-κB (NF-κB). However, its effects on the progression of endometriosis remain unsettled. The present study aimed to explore the *in vivo* alleviation of nobiletin on endometriosis and its mechanism of action. The mouse model of endometriosis was established and administered with nobiletin. The ectopic lesion size was measured and the hotplate test was performed to assess the amelioration of nobiletin on endometriosis. The expression of proliferation and angiogenesis relevant genes including proliferating cell nuclear antigen (PCNA), vascular endothelial growth factor (VEGF), and E-cadherin was measured by immunostaining and the mRNA expression of proinflammatory mediators including interleukin (IL)-6, IL-1β, tumor necrosis factor (TNF)-α, matrix metalloproteinases (MMP)-1, and MMP-3 was measured by RT-PCR. The change of NF-κB activity in endometriotic cells was evaluated by Western blotting and confirmed by luciferase assay. Administration of nobiletin significantly reduced lesions size and pain in endometriosis mice. Nobiletin significantly altered the expression of PCNA, VEGF, and E-cadherin in ectopic endometrium, as well as the levels of IL-6, IL-1β, TNF-α, MMP-1, and MMP-3. Nobiletin also showed remarkably impairment on the activation of NF-κB in promoting endometriotic cells, likely targeting on the activity of IκB kinases (IKKs). The present study provides the first evidence that nobiletin exerts protection on endometriosis via inhibition the activation of NF-κB, specifically on the activity of IκB kinases.

## Introduction

Endometriosis is defined as the presence of endometrial tissue outside the uterine cavity [1]. It is one of the most common gynecological disorders, affecting up to 10% of reproductive-aged women who suffer from chronic pelvic pain, dysmenorrhea, dyspareunia, and subfertility [2,3]. New discoveries on the genetic and immune systems in the endometrium of women with endometriosis, and the secreted cytokines of endometriotic lesions have given insights into the pathogenesis of this disorder, providing the foundation for new treatments for disease-associated pain and infertility [4]. It is widely accepted that local inflammation occurs in the peritoneal cavity of patients with endometriosis, which is characterized by the alteration of the immunologic components and inflammatory mediators in peritoneal fluid [5]. A number of studies have shown that hormone such as gonadotropin-releasing hormone (GnRH) agonists and progestogens reduced endometriotic lesion development. Anti-inflammatory drugs including tumor necrosis factor (TNF)-α inhibitors, peroxisome proliferator-activated receptor-g (PPAR-g) agonists, and antibody against matrix metalloproteinases (anti-MMP) exert therapeutic effects on endometriosis [6–8]. Along with this, the presence of nuclear factor-κB (NF-κB) in human endometrium supports its role in the physiology and pathophysiology of endometrial cells (ECs). Studies on the effects of NF-κB inhibitors in ECs and promoting endometriotic cells (EcCs) have shown that the inhibition of NF-κB could reduce endometriosis development and maintenance [9]. Aberrant cytokine levels in the peritoneal fluid, which is regulated by NF-κB pathway, result in a proinflammatory local environment, promoting survival and growth of EcC in endometriosis patients [5]. NF-κB is a transcriptional factor that plays a crucial role in inflammation, immunity, cell adhesion, invasion, cellular proliferation, apoptosis, and angiogenesis [10]. Usage of NF-κB inhibitory agents in experimental endometriosis models has highlighted the role of the NF-κB pathway in endometriosis initiation and progression, making this pathway to be an attractive target for the treatment and prevention of endometriosis.

Flavonoids have been reported to be functioned as anti-inflammatory agent and certain flavonoids affect stress/cytokine-induced NF-κB signal transduction. Among them, nobiletin is a polymethoxylated flavonoid found in citrus fruit peel and has been widely used as herb medicine for centuries, playing important roles in tumor suppression, immune stimulation, anti-inflammation, antioxidation, as well as some cardiovascular disease mitigation [11,12]. For example, nobiletin inhibits the expression of the allergic cytokines, interleukin (IL)-4 and TNF-α as well as the activation of their transcription factors NF-κB [13]. Given the inhibition of nobiletin on the NF-κB signaling which plays important roles in endometriosis pathogenesis, the objective of the present study was to investigate the effects of nobiletin on the development of endometriotic lesions and how the process is regulated by NF-κB in an *in vivo* experimental mice model of endometriosis.

## Materials and methods

### Ethics statement

All animal procedures for these experiments were approved by the Institutional Animal and Use Committee at Liaocheng People’s Hospital. All mice were housed within environmentally controlled temperature and at a 12/12 h (light/dark) schedule. All procedures were conducted in accordance with the approved animal protocol.

### Reagents

Nobiletin was isolated from *Citrus depressa* as described [14] and dissolved in saline containing 0.5% (v/v) Tween 80 (Sigma-Aldrich, PA, U.S.A.). The following primary antibodies were used: Primary antibodies used in immunohistochemistry against proliferating cell nuclear antigen (PCNA), vascular endothelial growth factor (VEGF), and E-cadherin were purchased from Santa Cruz Biotechnology (Santa Cruz, CA, U.S.A.). Rabbit monoclonal antibodies used in Western blotting against IκB kinase (IKK)α, p-IKKα, IκBα, p-IκBα, β-actin, and Lamin B were purchased from Abcam (Cambridge, MA, U.S.A.). Rabbit monoclonal antibody against p65 was purchased from Beyotime (AN365, Jiangsu, China). The horseradish peroxidase-conjugated secondary antibodies were purchased from Thermofisher Scientific (Rockville, MD, U.S.A.).

### Establishment of mouse model with endometriosis

Endometriosis was experimentally induced as previously described [15]. Briefly, C57BL/6 female mice at 3–4 weeks were injected subcutaneously with pregnant mare serum gonadotropin (PMSG, 2 IU per mouse; Sigma-Aldrich). Approximately 42–44 h post PMSG injection, uteri tissues were harvested from these donor mice. Uterine stroma and epithelium (endometrium) were carefully separated from myometrium under dissecting microscope and were cut into 1 mm^3^ pieces. Further, endometrial pieces were suspended in saline, and 400 µl of suspension was injected into the peritoneal cavity of the recipient mice at 8–10 weeks. Recipient mice (2- to 4-month-old wild-type C57BL/6 immunocompetent, reproductively intact females) were anesthetized with ketamine/xylazine (87.5 and 12.5 mg/kg body weight), and Carprofen (5 mg/kg body weight) was given as analgesic upon surgery conclusion immediately and 48 h post-operatively. According to our preliminary study, after the establishment of endometriosis, the animals were divided randomly into four groups (*n*=12): Sham control group, endometriosis (EM) group, EM plus nobiletin at 10 and 20 mg/kg groups. In the treatment group, mice were administered nobiletin intraperitoneally daily for 28 consecutive days, and then killed through cervical dislocation and the total surface area of ectopic lesions in each mouse was evaluated. Specifically, the abdominal cavity was immediately reopened, and all lesions were measured with two perpendicular diameters (the length D1 and the width D2). The surface area of each endometrial implant tissue was calculated by the formula: S = π × D1 × D2/4 (in mm^2^).

### Hotplate procedures

The sensitivity of the nociception was evaluated by the hotplate test [16] with a commercially available Hot Plate Analgesia Meter (Model BME-480, Tianjin, China) consisting of a metal plate of 25 × 25 cm in size, which can be heated to a constant temperature of 54.0 + 0.1°C. As described in the manufacture’s instruction, the latency to respond to thermal stimulus is defined to be the time length (in second) elapsed from the moment when the mouse was placed in the cylinder to the time when it licked hind paws or jumped off the hot plate. The latency was calculated as the mean of two readings recorded at intervals of 24 h. To assess the time-course progression in thermal latency as a result of induced endometriosis, mice went through hotplate procedure at day 3, 7, 14, 21, and 28 respectively.

### Immunohistochemistry

Mice ectopic lesions were fixed with 10% neutral buffered formalin for 24 h at room temperature, followed by immersion in 70% ethanol overnight at 4°C, then embedded in paraffin and sectioned at 4-μm thickness. For each paraffin-embedded tissue block, slide was stained by hematoxylin and eosin (H&E) and immunostained for different antibodies. For antigen retrieval, the slides were heated at 98°C in the EDTA buffer (pH 8.0) or the citric acid solution (pH 6.0) for a total of 30 min and cooled naturally at room temperature. The slides were incubated with primary antibodies against PCNA, VEGF, and E-cadherin, diluted to 1:2,000, 1:50, and 1:100 respectively at 4°C for 12 h. Further, after sufficient PBS wash, the slides were incubated with HRP-conjugated goat polyclonal antibody. The number and intensity of positive cells were counted by Image Pro-Plus 6.0 (Media Cybernetics, Inc., MD, U.S.A.). Images were obtained with the microscope fitted with a digital camera (Olympus, SD, U.S.A.).

### Quantitative real-time polymerase chain reaction (qRT-PCR)

Ectopic endometrial samples were collected from the pelvic lesion region, and mRNA was extracted with RNeasy Mini Kit (Qiagen, Germany) according to manufacturer’s instruction. Concentration and quality of total RNA were assessed with a NanoDrop 2000 spectrophotometer (ThermoFisher Scientific, DE, U.S.A.). RNA was reverse transcribed using cDNA synthesis kits (Takara, Dalian, China), the expression levels of IL-6, IL-1β, TNF-α, MMP-1, and MMP-3 were determined using Taqman Real-Time PCR assays (Thermofisher Scientific) on CFX96 Touch™ Real-Time PCR Detection System (Bio-Rad, CA, U.S.A.). The designed primers were as follows: β-actin, F: 5′-CTGGGACGACATGGAGAAAA-3′, R: 5′-AAGGAAGGCTGGAAGAGTGC-3′; TNF-α, F: 5′-GCCACCACGCTCTTCTGTC-3′, R: 5′-TGCTCCTCCACTTGGTGGTT-3′; IL-1β, F: 5′-TTGACGGACCCCAAAAGATG-3′, R: 5′-AGCTGCCACAGCTTCTCCAC-3′; IL-6, F: 5′-CCATCCAGTTGCCTTCTTGG-3′, R: 5′-TGCAAGTGCATCATCGTTGT-3′; MMP-1, F: 5′-GGACTCTGAGCTCTTCTACC-3′; R: 5′-CACTAGAGACAAGAGTGGC-3′; MMP-3, F: 5′-GGCAGAACCAAACAGGAGC-3′, R: 5′- GGCCCAGGAGTGCCTTCCCTCC-3′.

### Cell culture and treatment

Endometrial and endometriotic stromal cells were isolated and characterized according to previous studies [17]. Briefly, minced tissues were digested with type IV collagenase (Millipore, Shanghai, China), and stromal cells were separated from epithelial glands by passing through nylon meshes. The obtained stromal cells were then cultured in Dulbecco Modified Eagle Medium (Gibco, MD, U.S.A.) supplemented with 10% fetal bovine serum (FBS) and antibiotics at 37°C.

### Western blotting

The endometrial stromal cells were lysed and protein concentrations were determined by the Bradford method (Thermofisher Scientific, Gaithersburg, MD). Twenty-five micrograms of protein was loaded on 8% SDS-PAGE and transferred onto a polyvinylidene difluoride membrane (Millipore, Bedford, MA). Nonspecific binding was blocked in 5% nonfat milk at 4°C overnight. Membrane was then incubated with rabbit monoclonal antibodies against IKKα, p-IKKα, IκBα, p-IκBα, β-actin, P65, and Lamin B at a 1:1000 dilution for 1 h at 37°C. After washing with PBST buffer for three times, membrane was further incubated with HRP-conjugated second antibody at a 1:25,000 dilutions for 1 h at room temperature and detected by ECL substrate (Pierce, Rockville, MD).

### Luciferase assay

The HEK293 cells (2 × 10^5^) were seeded on 24-well plates (Corning, Shanghai, China) and transfected with p65 or IKKβ expression plasmids by standard calcium phosphate precipitation. After 34 h of transfection, cells were incubated with nobiletin of 10 and 20 µg/ml for 2 h respectively. Then, cells were harvested for luciferase assays. Luciferase assays were performed with a dual-specific luciferase assay kit (Promega, Durham, NC).

### Statistical analysis

The comparison of distributions of continuous variables between or among two or more groups was made using the *t*-test, as well as one-way and two-way ANOVA respectively. *P*-values less than 0.05 were considered statistically significant. All data were analyzed with GraphPad Prism 5.0 software (GraphPad, San Diego, CA, U.S.A.).

## Results

### Nobiletin reduced lesion size and hotplate latency in endometriosis mouse

**Figure 1:**
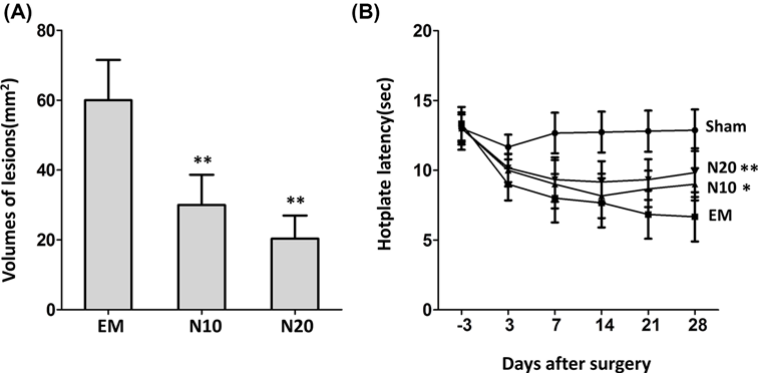
Nobiletin reduced lesions size and pain in endometriosis mouse (**A**) The average sizes of ectopic lesions were decreased by nobiletin dose dependently. (**B**) The time-course of the mean hotplate latency in different treatment groups. Abbreviations used in the figure: EM, endometriosis mice without treatment; N10, endometriosis mice treated 10 mg/kg/day; N20, endometriosis mice treated 20 mg/kg/day; *N*=6 mice per group. Values are expressed as mean ± S.E.M. of three independent experiments; *P<0.05 and ** *P*<0.01 vs. EM.

As shown in Figure 1A, we found nobiletin reduced lesion size dose dependently in endometriosis mouse. Specifically, the volume of the lesion in the endometriosis group is approximately 60 mm^2^, while the endometriosis mice treated with 10 mg/kg per day had lesion size down to 30 mm^2^, and even lower size of 20 mm^2^ in the endometriosis mice treated with 20 mg/kg per day. In fact, low- and high- dose of nobiletin treatment resulted in average of 50 and 67% reduction in lesion size respectively (*P*<0.01).

We also assessed hotplate responses of mice from 3 days prior to the surgical induction of endometriosis or sham surgery until 4 weeks after the surgery with nobiletin treatment (Figure 1B). There were no differences in hotplate latency among all groups prior to the surgery, indicating the matched selection of mice in the experiment. However, starting from 3 days after surgery, the hotplate latencies decreased in all four groups due to the surgical trauma and adhesion, and the hotplate latencies in endometriosis groups were significantly lower than the one in the Sham surgery (no endometriosis). This trend has been kept to 7 weeks after the surgery. Of note, nobiletin treatment at both low- and high- doses significantly recovered the hotplate latency compared with the endometriosis mice without treatment, indicating the pain reduce in treated groups.

### Effects of nobiletin on PCNA, VEGF, and E-cadherin immunochemistry in ectopic endometrium in endometriosis mouse

**Figure 2:**
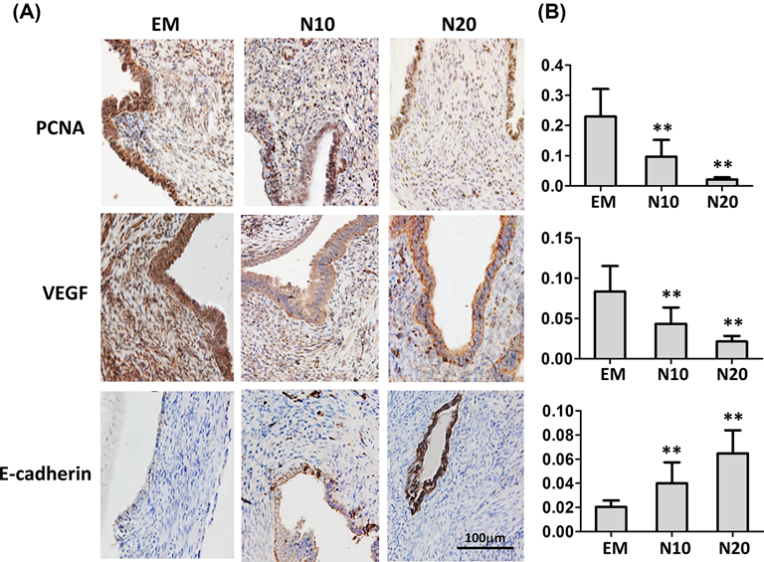
Effects of nobiletin on the expression of proliferation and angiogenesis relevant genes in ectopic endometrium *in vivo* (**A**) Immunohistochemistry analysis of PCNA, VEGF, and E-cadherin. (**B**) The staining levels of PCNA, VEGF, and E-cadherin in different treatment groups. Abbreviations used in the figure: EM, endometriosis mice without treatment; N10, endometriosis mice treated 10 mg/kg/day; N20, endometriosis mice treated 20 mg/kg/day; *N*=6 mice per group. Values are expressed as means ± S.E.M. of three independent experiments; **P<0.01 vs. EM.

Figure 2A shows the PCNA, VEGF, and E-cadherin immunostaining in ectopic endometrium in mice with endometriosis. It can be seen that the staining levels of PCNA, VEGF, and E-cadherin vary significantly among different groups (Figure 2B). In particular, mice treated with nobiletin had significantly lower immunostaining to PCNA and VEGF in both low- and high-dose of nobiletin treated groups (*P*<0.01), and higher staining level of E-cadherin in nobiletin treated group, as compared with the untreated mice (*P*<0.01).

### Inhibition of nobiletin on inflammatory responses in endometriosis mouse

**Figure 3:**
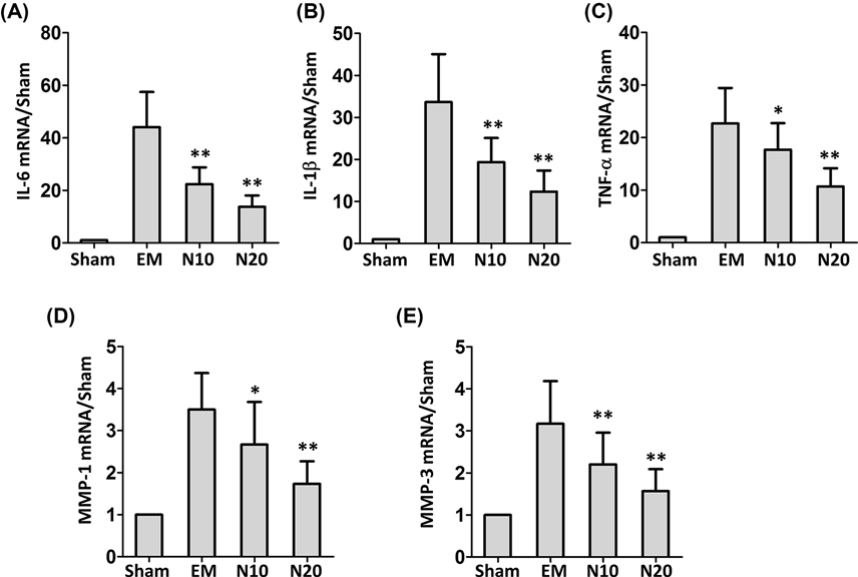
Effects of nobiletin on inflammatory responses in endometriosis mouse (**A**–**E**) mRNA levels of IL-6, IL-1β, TNF-α, MMP-1, and MMP-3 in different treatment groups. Abbreviations used in the figure: EM, endometriosis mice without treatment; N10, endometriosis mice treated 10 mg/Kg/day; N20, endometriosis mice treated 20 mg/kg/day; *N*=6 mice per group. Values are expressed as means ± S.E.M. of three independent experiments; *P<0.05 and **P<0.01 vs. EM.

As indicated in Figure 3A–E, the mRNA levels of IL-6, IL-1β, TNF-α, MMP-1, and MMP-3 in different treatment groups were evaluated by RT-PCR. Compared with the sham group, the expression levels of all these inflammatory factors were significant elevated in the endometriosis mice, suggesting the inflammation caused by the surgery. As expected, the treatment with nobiletin at 10 mg/kg/day decreased the levels of IL-6, IL-1β, and MMP-3 by 41, 30 and 33% respectively (*P*<0.01) and reduced the levels of TNF-α and MMP-1 by 18 and 24% respectively (*P*<0.05). Consistently, the treatment with nobiletin at 20 mg/kg/day reduced the levels of IL-6, IL-1β, TNF-α, MMP-1, and MMP-3 by 57, 66, 21, 30 and 47% respectively<0.01).

### Impact of nobiletin on NF-κB pathway activation

**Figure 4:**
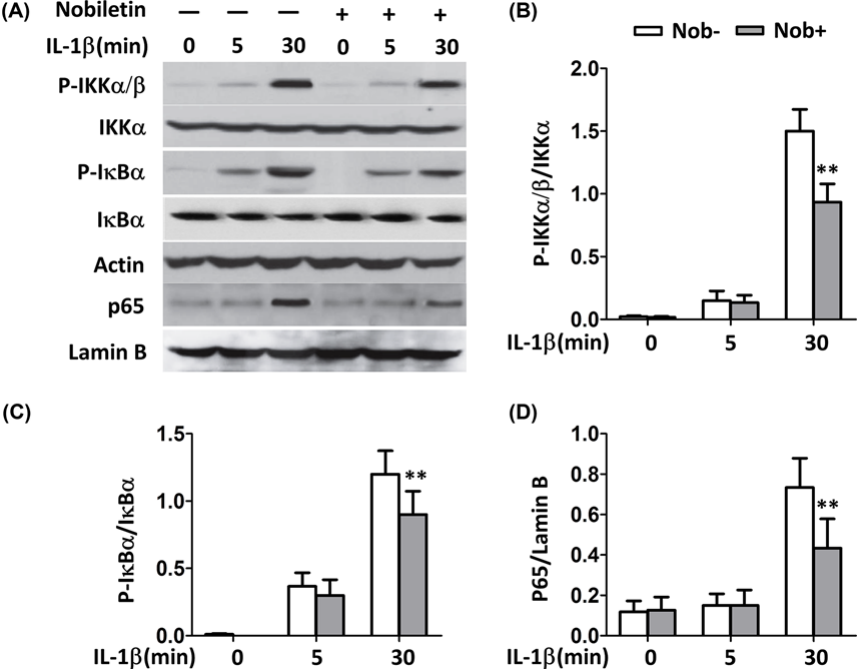
Activation of NF-κB is impaired in ESCs in response to different nobiletin concentration (**A**) IB analyses of the indicated phosphorylated (P-) and total proteins in whole-cell lysates or cytoplasmic and nuclear (p65) extracts of ESCs stimulated with IL-1β for the indicated time periods with (Nob+, 20 μg/ml) or without (Nob-) nobiletin. (**B–D**) The bar graph showed the intensities of genes expression. Values are expressed as means ± S.E.M. of three independent experiments; **P<0.01 vs.Nob- group.

To examine whether NF-κB pathway is involved in the antagonism of nobiletin on endometriosis, IKKα/β activation and IκBα phosphorylation were evaluated by immunoblotting after stimulation by IL-1β. NF-κB complex (p50 and p65), which is associated with IκB to retain in the cytosol under resting state, is released and translocates to the nucleus [18]. Therefore, we first investigated the time-course of IκBα phosphorylation in promoting endometriotic cells. Cells were treated by IL-1β (10 ng/ml) for 5 and 30 min and total proteins were extracted for Western blotting detection. The results showed that treatment with IL-1β enhanced the phosphorylation of IKKα and IκBα time dependently in human endometrial cells (Figure 4A–C). The phosphorylation of nuclear extract (p65) is also enhanced by IL-1β stimulation. With the treatment of nobiletin, the phosphorylation of these factors was reduced significantly at 30 min. Since p65 subunit is responsible for the transcriptional activity of NF-κB [19], we also determined the p65 nuclear accumulation. As illustrated in Figure 4D, nobiletin was able to inhibit the levels of p65 in time-dependent manner.

### Suppression of nobiletin on IKK-mediated activation of NF-κB pathway

**Figure 5:**
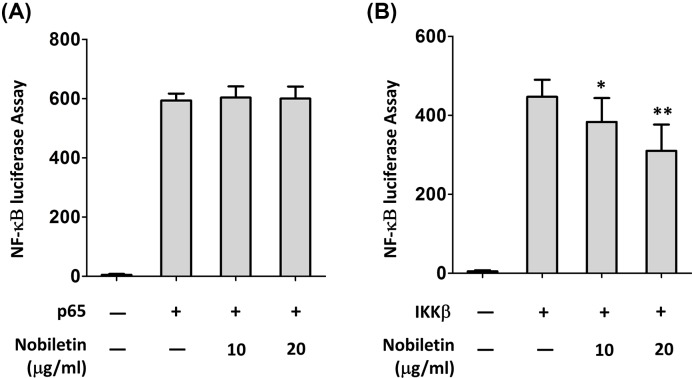
Nobiletin inhibited the activity of IKKs but did not inhibit p65 (**A** and **B**) Cells were incubated with nobiletin respectively at 10 and 20 μg/ml for 2 h before harvest. Luciferase assays were performed 36 h after transfection; *P<0.05 and **P<0.01 vs. IKKβ+/Nob- group.

To detect the effects of nobiletin on NF-κB dependent gene expression, HEK293 cells were transiently transfected with p65 and IKKβ promoter-dependent luciferase reporter construct. Treatment with nobiletin did not show change on p65 expression levels (Figure 5A), while the expression of IKKβ was repressed by pretreatment of nobiletin at low- and high-doses with comparing with control (Figure 5B).

## Discussion

Endometriosis was first described by Daniel Shroen in 1690 [20] and is considered to be an inflammation type. Immune and inflammatory reactions at the cellular and molecular levels in endometriosis could contribute to the endometriotic implant survival and growth, and thus cause chronic pain and affect fertility [21]. The NF-κB system dysfunction indicated by the molecular alterations of IL-6 and RelA (p65) during the late secretory phase in eutopic endometrium from endometriosis patients suggested that NF-κB could be an important factor in endometriosis etiology [22].

Nobiletin is polymethoxy flavonoid that is abundant in the pericarp of Citrus. Nobiletin exhibits various biological activities, including anti-inflammatory and antioxidative effects. Citrus aurantium extract, which is rich in nobiletin has been reported to activate lipid metabolism related genes, thus ameliorating ethanol-induced liver injury in mice [23]. Nobiletin attenuates lipopolysaccharide/D galactosamine induced mouse model partly by inhibiting cytokine production mediated by NF-κB [24]. Nobiletin ameliorate scratching in histamine induced mice by inhibited vascular permeability, the expression of IL-4 and TNF-α as well as the activation of NF-κB [13]. To our knowledge, there are no published data on the treatment of nobiletin in the case of endometriosis. To explore the medical usage of nobiletin and better understand the mechanisms underlying the alleviation of endometriosis by nobiletin, we administrated nobiletin in mouse model with endometriosis and the expression of the relevant inflammatory factors and the activation of the NF-κB pathway were evaluated as compared with controls.

It is well accepted that endometriotic lesions require new blood supply to survive under the hypoxic environment of ectopic sites, therefore angiogenesis is essential for the development and progression of endometriosis [25]. VEGF expression has been reported to be elevated in the eutopic glandular epithelium and peritoneal fluid of women with endometriosis during the late secretory phase [26,27]. In our study, we found nobiletin reduced lesion size in dose-dependent manner in endometriosis mouse, and we further detected how nobiletin affects the angiogenic ability with the endometriotic lesions. Compared with the untreated endometriosis mice, treatment with nobiletin significantly lowered PCNA and VEGF immunostaining, and showed higher staining level of E-cadherin in both low- and high-dose of nobiletin treated groups (*P*<0.01). The expression of several angiogenic factors is regulated by NF-κB, for instance, macrophages have been reported to produce VEGF under the control of NF-κB activation [28]. It is therefore possible that nobiletin inhibited neoangiogenesis in endometriosis is a NF-κB -dependent process.

NF-κB is a transcription factor involved in numerous pathologies and known to be a proinflammatory, mitogenic, and antiapoptotic factor in many cell types [10]. Its function was recognized in tumor necrosis factor and IL-1β receptors signaling pathways in several cell types [29]. Human endometrial cells have been shown to express NF-κB proteins [29,30] and to activate NF-κB in response to IL-1β [31]. Altered expression of proinflammatory cytokines in the endometrium has been shown to be associated with female fertility reduction and abortion dysregulation [32,33]. It has been suggested that the level of IL-6 in serum and TNF-α in peritoneal fluid could be used as markers to discriminate patients with endometriosis from others [34,35]. Our results showed that the treatment with nobiletin significantly decreased the gene expression levels of IL-6, IL-1β and MMP-3 (*P*<0.01), TNF-α and MMP-1 (*P*<0.05). Due to the important participation of these factors in the pathophysiology of endometriosis, nobiletin could contribute to suppress the establishment and proliferation of ectopic endometrial implants.

NF-κB is composed of homodimers and heterodimers of five members including p65 (RelA), p105/p50, p100/p52, RelB, and c-Rel. The classic form of NF-κB is the heterodimer of the p50 and p65 subunits [6]. In response to multiple stimuli, the IKKs complex binds with the regulatory subunit IKKγ/NF-κB essential modulator, which subsequently forms the TNF-α receptor complex and promotes IκB phosphorylation. Phosphorylated IκBα is rapidly ubiquitinated and degraded via a proteasome pathway. Degradation of IκBα leads to the expression of NF-κB, which translocates into the nucleus where it binds to specific binding sites within the promoter regions of target genes [36].

Chemotherapy agents or acute stimuli such as TNF-α, LPS, or PMA lead to the activation of IKKs which in turn phosphorylate two key serine residues, Ser32 and Ser36, on IκBs within the N-terminal response domain [7]. These products are involved in the initiation, maintenance, and progression of endometriosis by inducing endometrial fragment adhesion, proliferation, and neovascularization [37]. The immunocytochemical analysis in cultured epithelial endometrial cells revealed that the staining intensity of p65 was low during the proliferative phase, but increased during the secretory phase and was maximal at the time of implantation [29]. In the present study, we investigated the time-course of IκBa phosphorylation in IL-1β mediated endometriotic cells, and our results showed that the phosphorylation of IKKα, IκBα, and p65 was reduced significantly at 30 min by nobiletin treatment. Further, to detect the effect of nobiletin on NF-κB dependent gene expression, p65 and IKKβ promoter-dependent luciferase reporter constructs were transfected into HEK293 cells, and we found nobiletin repressed the expression of IKKβ rather than p65, suggesting that IKK activation but not NF-κB complex.

To this end, we tested the effects of nobiletin on the development of endometriosis induced in mice and found that nobiletin decreased lesion sizes and pain in the animals. Further, we found that this alleviation was realized through the down-regulation of NF-κB signaling pathway and supported the reduction of the angiogenic and inflammatory gene expression, as well as the NF-κB complex activation.

## Conclusion

The present study provides the first evidence that nobiletin exerts protection on endometriosis via inhibition the activation of NF-κB, specifically on the activity of IκB kinases.

## Abbreviations

EC: endometrial cell
EcC: endometriotic cell
ECL: enhanced chemiluminescene
EM: endometriosis
GnRH: gonadotropin-releasing hormone
H&E: hematoxylin and eosin
HRP: horseradish peroxidase
IKK: IκB kinase
IL: interleukin
MMP: matrix metalloproteinases
NF-κB: nuclear factor-κB
PCNA: proliferating cell nuclear antigen
PMSG: pregnant mare serum gonadotropin
qRT-PCR: quantitative real-time polymerase chain reaction
TNF: tumor necrosis factor
VEGF: vascular endothelial growth factor

## References

[1] Defrere S., Gonzalez-Ramos R., Lousse J.C., Colette S., Donnez O., Donnez J. (2011) Insights into iron and nuclear factor-kappa B (NF-kappaB) involvement in chronic inflammatory processes in peritoneal endometriosis. Histol. Histopathol. 26, 1083–1092 21692040 10.14670/HH-26.1083

[2] Holoch K.J. and Lessey B.A. (2010) Endometriosis and infertility. Clin. Obstet. Gynecol. 53, 429–438 10.1097/GRF.0b013e3181db7d71 20436320

[3] Nasu K., Nishida M., Ueda T., Yuge A., Takai N. and Narahara H. (2007) Application of the nuclear factor-kappaB inhibitor BAY 11-7085 for the treatment of endometriosis: an in vitro study. Am. J. Physiol. Endocrinol. Metab. 293, E16–E23 10.1152/ajpendo.00135.2006 16896168

[4] Giudice L.C. and Kao L.C. (2004) Endometriosis. Lancet 364, 1789–1799 10.1016/S0140-6736(04)17403-5 15541453

[5] Lousse J.C., Van Langendonckt A., Gonzalez-Ramos R., Defrere S., Renkin E. and Donnez J. (2008) Increased activation of nuclear factor-kappa B (NF-kappaB) in isolated peritoneal macrophages of patients with endometriosis. Fertil. Steril. 90, 217–220 10.1016/j.fertnstert.2007.06.015 17889859

[6] Bruner K.L., Matrisian L.M., Rodgers W.H., Gorstein F. and Osteen K.G. (1997) Suppression of matrix metalloproteinases inhibits establishment of ectopic lesions by human endometrium in nude mice. J. Clin. Invest. 99, 2851–2857 10.1172/JCI119478 9185507 PMC508135

[7] Falconer H., Mwenda J.M., Chai D.C., Wagner C., Song X.Y., Mihalyi A. (2006) Treatment with anti-TNF monoclonal antibody (c5N) reduces the extent of induced endometriosis in the baboon. Hum. Reprod. 21, 1856–1862 10.1093/humrep/del044 16517562

[8] Mihalyi A., Simsa P., Mutinda K.C., Meuleman C., Mwenda J.M. and D’Hooghe T.M. (2006) Emerging drugs in endometriosis. Expert Opin. Emerg. Drugs 11, 503–524 10.1517/14728214.11.3.503 16939388

[9] Gonzalez-Ramos R., Van Langendonckt A., Defrere S., Lousse J.C., Colette S., Devoto L. (2010) Involvement of the nuclear factor-kappaB pathway in the pathogenesis of endometriosis. Fertil. Steril. 94, 1985–1994 10.1016/j.fertnstert.2010.01.013 20188363

[10] Viatour P., Merville M.P., Bours V. and Chariot A. (2005) Phosphorylation of NF-kappaB and IkappaB proteins: implications in cancer and inflammation. Trends Biochem. Sci. 30, 43–52 10.1016/j.tibs.2004.11.009 15653325

[11] Knekt P., Jarvinen R., Seppanen R., Hellovaara M., Teppo L., Pukkala E. (1997) Dietary flavonoids and the risk of lung cancer and other malignant neoplasms. Am. J. Epidemiol. 146, 223–230 10.1093/oxfordjournals.aje.a009257 9247006

[12] Li S., Yu H. and Ho C.T. (2006) Nobiletin: efficient and large quantity isolation from orange peel extract. Biomed. Chromatogr. 20, 133–138 10.1002/bmc.540 15999338

[13] Jang S.E., Ryu K.R., Park S.H., Chung S., Teruya Y., Han M.J. (2013) Nobiletin and tangeretin ameliorate scratching behavior in mice by inhibiting the action of histamine and the activation of NF-kappaB, AP-1 and p38. Int. Immunopharmacol. 17, 502–507 10.1016/j.intimp.2013.07.012 23938254

[14] Nagase H., Yamakuni T., Matsuzaki K., Maruyama Y., Kasahara J., Hinohara Y. (2005) Mechanism of neurotrophic action of nobiletin in PC12D cells. Biochemistry 44, 13683–13691 10.1021/bi050643x 16229458

[15] Nothnick W.B., Graham A., Holbert J. and Weiss M.J. (2014) miR-451 deficiency is associated with altered endometrial fibrinogen alpha chain expression and reduced endometriotic implant establishment in an experimental mouse model. PLoS One 9, e100336 10.1371/journal.pone.0100336 24937656 PMC4061076

[16] Bannon A.W. and Malmberg A.B. (2007) Models of nociception: hot-plate, tail-flick, and formalin tests in rodents. Curr. Protoc. 41, 8.9.1–8.9.16 10.1002/0471142301.ns0809s41 18428666

[17] Hsiao K.Y., Chang N., Lin S.C., Li Y.H. and Wu M.H. (2014) Inhibition of dual specificity phosphatase-2 by hypoxia promotes interleukin-8-mediated angiogenesis in endometriosis. Hum. Reprod. 29, 2747–2755 10.1093/humrep/deu255 25316445

[18] Perkins N.D. (2007) Integrating cell-signalling pathways with NF-kappaB and IKK function. Nat. Rev. Mol. Cell Biol. 8, 49–62 10.1038/nrm2083 17183360

[19] Tse A.K., Wan C.K., Shen X.L., Yang M. and Fong W.F. (2005) Honokiol inhibits TNF-alpha-stimulated NF-kappaB activation and NF-kappaB-regulated gene expression through suppression of IKK activation. Biochem. Pharmacol. 70, 1443–1457 10.1016/j.bcp.2005.08.011 16181613

[20] Knapp V.J. (1999) How old is endometriosis? Late 17th- and 18th-century European descriptions of the disease Fertil. Steril. 72, 10–14 10428141 10.1016/s0015-0282(99)00196-x

[21] Kaponis A., Iwabe T., Taniguchi F., Ito M., Deura I., Decavalas G. (2012) The role of NF-kappaB in endometriosis. Front. Biosci. 4, 1213–1234 22652867 10.2741/s327

[22] Ponce C., Torres M., Galleguillos C., Sovino H., Boric M.A., Fuentes A. (2009) Nuclear factor kappaB pathway and interleukin-6 are affected in eutopic endometrium of women with endometriosis. Reproduction 137, 727–737 10.1530/REP-08-0407 19129371

[23] Choi B.K., Kim T.W., Lee D.R., Jung W.H., Lim J.H., Jung J.Y. (2015) A polymethoxy flavonoids-rich Citrus aurantium extract ameliorates ethanol-induced liver injury through modulation of AMPK and Nrf2-related signals in a binge drinking mouse model. Phytother. Res. 29, 1577–1584 10.1002/ptr.5415 26178909

[24] He Z., Li X., Chen H., He K., Liu Y., Gong J. (2016) Nobiletin attenuates lipopolysaccharide/Dgalactosamineinduced liver injury in mice by activating the Nrf2 antioxidant pathway and subsequently inhibiting NFkappaBmediated cytokine production. Mol. Med. Rep. 14, 5595–5600 10.3892/mmr.2016.5943 27878238

[25] Rocha A.L., Reis F.M. and Taylor R.N. (2013) Angiogenesis and endometriosis. Obstet. Gynecol. Int. 2013, 859619 10.1155/2013/859619 23766765 PMC3677669

[26] Donnez J., Smoes P., Gillerot S., Casanas-Roux F. and Nisolle M. (1998) Vascular endothelial growth factor (VEGF) in endometriosis. Hum. Reprod. 13, 1686–1690 10.1093/humrep/13.6.1686 9688413

[27] Kupker W., Schultze-Mosgau A. and Diedrich K. (1998) Paracrine changes in the peritoneal environment of women with endometriosis. Hum. Reprod. Update 4, 719–723 10.1093/humupd/4.5.719 10027625

[28] Kiriakidis S., Andreakos E., Monaco C., Foxwell B., Feldmann M. and Paleolog E. (2003) VEGF expression in human macrophages is NF-kappaB-dependent: studies using adenoviruses expressing the endogenous NF-kappaB inhibitor IkappaBalpha and a kinase-defective form of the IkappaB kinase 2. J. Cell Sci. 116, 665–674 10.1242/jcs.00286 12538767

[29] Laird S.M., Tuckerman E.M., Cork B.A. and Li T.C. (2000) Expression of nuclear factor kappa B in human endometrium; role in the control of interleukin 6 and leukaemia inhibitory factor production. Mol. Hum. Reprod. 6, 34–40 10.1093/molehr/6.1.34 10611258

[30] Page M., Tuckerman E.M., Li T.C. and Laird S.M. (2002) Expression of nuclear factor kappa B components in human endometrium. J. Reprod. Immunol. 54, 1–13 10.1016/S0165-0378(01)00122-X 11839392

[31] Cao W.G., Morin M., Metz C., Maheux R. and Akoum A. (2005) Stimulation of macrophage migration inhibitory factor expression in endometrial stromal cells by interleukin 1, beta involving the nuclear transcription factor NFkappaB. Biol. Reprod. 73, 565–570 10.1095/biolreprod.104.038331 15901641

[32] von Wolff M., Thaler C.J., Strowitzki T., Broome J., Stolz W. and Tabibzadeh S. (2000) Regulated expression of cytokines in human endometrium throughout the menstrual cycle: dysregulation in habitual abortion. Mol. Hum. Reprod. 6, 627–634 10.1093/molehr/6.7.627 10871650

[33] Kao L.C., Germeyer A., Tulac S., Lobo S., Yang J.P., Taylor R.N. (2003) Expression profiling of endometrium from women with endometriosis reveals candidate genes for disease-based implantation failure and infertility. Endocrinology 144, 2870–2881 10.1210/en.2003-0043 12810542

[34] Bedaiwy M.A., Falcone T., Sharma R.K., Goldberg J.M., Attaran M., Nelson D.R. (2002) Prediction of endometriosis with serum and peritoneal fluid markers: a prospective controlled trial. Hum. Reprod. 17, 426–431 10.1093/humrep/17.2.426 11821289

[35] Cheong Y.C., Shelton J.B., Laird S.M., Richmond M., Kudesia G., Li T.C. (2002) IL-1, IL-6 and TNF-alpha concentrations in the peritoneal fluid of women with pelvic adhesions. Hum. Reprod. 17, 69–75 10.1093/humrep/17.1.69 11756364

[36] Hoesel B. and Schmid J.A. (2013) The complexity of NF-kappaB signaling in inflammation and cancer. Mol. Cancer 12, 86 10.1186/1476-4598-12-86 23915189 PMC3750319

[37] Lebovic D.I., Mueller M.D. and Taylor R.N. (2001) Immunobiology of endometriosis. Fertil. Steril. 75, 1–10 10.1016/S0015-0282(00)01630-7 11163805

